# A rare complication of catherer insertion pericardiocentesis

**DOI:** 10.11604/pamj.2017.26.5.11494

**Published:** 2017-01-04

**Authors:** Spyridon Davakis, Christos Damaskos

**Affiliations:** 1 First Department of Surgery, Laiko General Hospital, National and Kapodistrian University of Athens, Athens Greece; 2 Second Department of Propedeutic Surgery, Laiko General Hospital, National and Kapodistrian University of Athens, Athens Greece; 3 Laboratory of Experimental Surgery and Surgical Research N.S. Christeas, Medical School, National and Kapodistrian University of Athens, Athens, Greece

**Keywords:** Pericadriocentesis, catheter, complication

## Image in medicine

Pericardiocentesis with catheter insertion for pericardial drainage is a common procedure used for the treatment of pericardial effusions and of cardiac tamponade as well. The most commonly described complications of an indwelling pericardial catheter system are catheter blockage and infection. We present a rare case of a 63 years old patient with a pericardial catheter for pericardial effusion drainage. He was presented with elevated body temperature and chills, a day after a pericardial catheter insertion. His clinical examination did not reveal any particular findings, while the catheter had stop draining two days earlier. His laboratory examinations revealed slightly increased WBCs' (13.300/ml). Following that, a chest x-ray examination revealed winding of the catheter along the pericardium. Under general anesthesia, the patient underwent left thoracotomy; the pericardial catheter was found tight wreathed and infiltrated to the inner coat of the pericardium. The catheter was carefully removed and a pericardial window was then performed. His post-operative course was uneventful, and he was discharged five days later.

**Figure 1:**
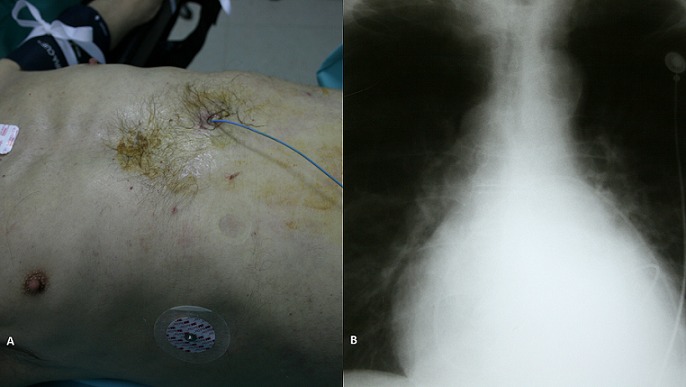
(A) pericardiocentisis with catheter insertion; (B) chest X-ray showing pericardial catheter winded to the pericardium

